# Mixed-phenotype acute leukemia: Flow cytometric analysis in a tertiary care center of Bangladesh

**DOI:** 10.1016/j.lrr.2026.100612

**Published:** 2026-09-23

**Authors:** Shirin Tarafder, Sheikh Anisul Haque

**Affiliations:** a Department of Microbiology and Immunology, Bangladesh Medical university, Dhaka, Bangladesh; b Department of Transfusion Medicine, Khwaja Yunus Ali Medical College and Hospital, Sirajganj, Bangladesh

**Keywords:** MPAL, Acute leukemia, Flow cytometry, Immunophenotyping, WHO

## Abstract

**Background:**

Mixed-phenotype acute leukemia (MPAL) is a rare but difficult to treat hematologic malignancy.

**Methods:**

flow cytometry immunophenotyping method was used in EDTA bone marrow and/or peripheral blood taken from all consecutive morphologically diagnosed acute leukemia (AL) patients from November 2015 to December 2019.

**Results:**

Among 616 consecutive AL cases diagnosed and evaluated during this period, 22(3.57%) patients with MPAL were identified fulfilling WHO 2008 criteria for immunophenotypic characteristics of AL. Fifteen (68.2%) cases were diagnosed as B/myeloid, 5(22.72%) as T/myeloid and 2 (9.1%) as B/T MPAL. Morphologically diagnosed 15 (68.2%) AML cases were immunophenotypically B/Myeloid MPAL (12/15) and 3/15 cases were T/Myeloid MPAL. Morphologically 7 (31.8%) were ALL of which immunophenotypically 3/7 cases were B/Myeloid MPAL, 2/7 cases were T/Myeloid MPAL and 2/7 were B/T MPAL.

**Conclusion:**

Multiparametric flow cytometry using comprehensive panel of monoclonal antibodies is a valuable tool to diagnose MPAL known to have a poor outcome.

## Highlights

• B/Myeloid MPAL is more common subtype.
• Flowcytometry can differentiate MPAL from Acute Leukemia with aberrant antigen expression.
• Flowcytometry is a valuable tool to diagnose MPAL.
• There is marked male predominance (male: female= 2.7:1).

## 1 Introduction

Mixed-phenotype acute leukemia (MPAL) is a rare but difficult to treat hematologic malignancy with immunophenotypic co-expression of at least two cell lineages, or with only rare cases involving all three lineages, e.g. B/Myeloid, T/Myeloid, B/T, and occasional trilineage Myeloid/B/T [1]. MPAL is a rare disease, representing only 3–5% of acute leukemias of all age groups, and 2.4–3.7% in children [2]. It affects both adults and children, more frequently adults and has slight male preference. The prognosis for MPAL is poor comparing to other acute leukemias, with an overall survival of 18 months [3,4]. According to 2008 World Health Organization (WHO) classification, these cases are identified as MPAL, under the category of acute leukemias of ambiguous lineage [3]. MPAL encompasses leukemias containing separate populations of blasts of more than one lineage (bilineal or bilineage), and a single population of blasts co-expressing antigens of more than one lineage (biphenotypic acute leukemia, BAL) [3]. The B/Myeloid MPAL is the most common among MPALs, followed by T/Myeloid, and other rare types of MPALs [4]. The 2008 World Health Organization classification established strict criteria for diagnosis of mixed phenotype acute leukemia, emphasizing myeloperoxidase for myeloid lineage assignment, cytoplasmic CD3 for T lineage assignment, and CD19 and other B cell markers for B lineage assignment [5]. Extensive immunophenotyping is necessary to confirm that the cells indeed belong to 2 different lineages or coexpress diferrentiation antigens of more than 1 lineage [1].

The morphological diagnosis of acute leukemia in the present study was done according to the French-American-British (FAB) classification during the original clinical evaluation. The objective of this retrospective study was to characterize morphologically diagnosed acute leukemia cases by multiparametric flow cytometry and diagnose MPAL following the WHO 2008 criteria. The present study could not apply contemporary molecularly integrated classification as this study was conducted retrospectively using cases between 2015 and 2019, where comprehensive molecular and cytogenetic data were not available. Therefore, the present study focuses on the identification and immunophenotypic characterization of MPAL using flow cytometry within the WHO 2008 diagnostic criteria and to study the incidence, clinical features, hematological profile and immunophenotypic expression profile of MPAL at our center.

## 2 Materials and methods

### 2.1 Study place and sample selection

A retrospective study was done by obtaining data from flow cytometry laboratory of Microbiology and Immunology department of our institute, where peripheral blood or bone marrow specimens obtained from 616 patients were submitted for MFC immunophenotyping and cell morphology by peripheral blood or bone marrow smear (PBS) from November 2015 to December 2019. Data were extracted from the database and anonymized.

### 2.2 Study procedure

The study was done on data obtained from 616 patients whose peripheral blood or bone marrow specimens were submitted for MFC immunophenotyping and cell morphology by peripheral blood smear (PBS). The clinical indications for MFC analysis were clinical features and hematological profile suggestive of acute leukemia done by Hemato Oncolgists. The peripheral blood smear were stained by Giemsa staining. The morphological diagnosis was incorporated with MFC analysis, reviewed and categorized them into Mixed-phenotype acute leukemia (MPAL) and acute leukemia categories.

The morphological diagnosis of acute leukemia in the present study was done according to the French-American-British (FAB) classification during the original clinical evaluation. For all patients molecular and cytogenetic investigations were not available. Therefore, retrospective reclassification was not feasible.

Four color MFC immunophenotyping was performed using a Beckman Coulter Cytomics FC500 flow cytometer using software CXP to analyze data. The following fluorescent monoclonal antibodies were used: CD45, CD34,TdT, HLA-DR, Anti-MPO, CD13,CD33, CD117, CD3, CD5,CD7,CD19, cytoplasmic CD79a, CD22, CD10. The antibodies were conjugated with fluorescein isothiocyanate (FITC), phycoerythrin (PE), PerCP- Cy5.5, and R Phycoerythrin-Texas Red-X (ECD). Surface and cytoplasmic staining was performed according to the manufacturer's recommended procedures, including red blood cell lysis and permeabilization for cytoplasmic antigen detection. CD45/side-scatter gating and additional scatter- and antigen-based gating strategies were used to identify the blast population.

A total of 30,000 events were acquired in target gate. Any antigen marker was considered positive if 20% or more of the cells reacted with a particular antibody, which was used as an operational laboratory cutoff for interpretation of the flow cytometric data. For lineage assignment the overall immunophenotypic pattern, lineage-associated antigen combinations and the WHO 2008 criteria for MPAL were considered. MPO was used as lineage specific myeloid differentiation marker and cytoplasmic CD3 (cCD3) was used as lineage specific T-cell differentiation marker. Cases classified as T/myeloid MPAL showed cCD3 positivity in >20% of blasts together with MPO expression and additional T-lineage markers including CD5 and CD7. B/myeloid cases expressed MPO together with B-lineage markers, including CD19 and cytoplasmic CD79a.

The study was performed during 2015-2019 using a four-color platform, the methodology could not incorporate contemporary 8-10 color or higher-color flow cytometry because of its unavailability. The methodological limitations were therefore taken into consideration when interpreting the immunophenotypic findings.

MPAL cases were classified according to the World Health Organization (WHO) 2008 classification criteria, which were applicable during the study period (November 2015 to December 2019). Primarily the diagnosis was based on morphological findings and multiparametric flow cytometric immunophenotyping. For all patients molecular and cytogenetic investigations were not available. Therefore, specific genetic abnormalities such as BCR::ABL1 or KMT2A rearrangements, could not be retrospectively assigned in this study. Therefore, the present study should be considered an immunophenotypic characterization of MPAL using the WHO 2008 criteria.

Clinical manifestations and all the information were recorded properly in the data collection sheet. Confidetiality were maintained. Institutional permission was obtained to conduct this retrospective study. This study was approved by the Ethics Review Board of Bangladesh Bioethics Society***.*** Data were extracted from the database and anonymized. A password protected folder was created to store the data.

### 2.3 Data analysis

Data were analyzed using IBM SPSS Statistics for Windows, Version 27.0 (IBM Corp., Armonk, NY, USA). Continuous variables were expressed as mean ± standard deviation or median with range, as appropriate. Normality was assessed using visual inspection and distribution characteristics. As blast percentage was not normally distributed, comparison between AML and ALL groups was performed using the Mann–Whitney U test. Categorical variables were expressed as frequencies and percentages, and group comparisons were performed using Fisher’s exact test due to small cell counts. FAB subtype and immunophenotype distributions were analyzed descriptively without inferential testing. A *p* value <0.05 was considered statistically significant.

## 3 Results

### 3.1 Immunophenotypic diagnosis and distribution of Acute leukemia and Mixed phenotype acute leukemia cases

Among 616 consecutive Leukemia cases diagnosed and evaluated during this period, 594(96.43%) cases as acute leukemia (AL) and 22 (3.57%) patients with MPAL were identified fulfilling World Health Organization (WHO) 2008 criteria for immunophenotypic characteristics of AL (Supplementary Fig. 1). Of 22 MPAL cases 15(68.2%) cases were diagnosed as B/myeloid, 5(22.7%) as T/myeloid and 2 (9.1%) as B/T MPAL (Table 1; Supplementary Fig. 1).

**Table 1:** 95% confidence intervals (CIs) for the key proportions of MPAL immunophenotypic subtypes (*N* = 22).

MPAL immunophenotype	n/N	Proportion	95% CI
B/myeloid	15/22	68.2%	**45.1–86.1%**
T/myeloid	5/22	22.7%	**7.8–45.4%**
B/T	2/22	9.1%	**1.1–29.2%**

{exact (Clopper–Pearson) 95% confidence intervals}.

### 3.2 Patient characteristics

Of the 22 MPAL cases 16 (72.73%) were male and 6 (27.27%)were female, giving an overall male-to-female ratio of approximately 2.7:1. Fourteen patients were adults and had a male- to- female ratio of 5:2, whereas 8 patients were children and had a male- to- female ratio of 3:1. For this study, patients aged <18 years were considered children and those aged ≥18 years were considered adults. Median age of this cohort (*n* = 22) was 23.5 years (range: 1 -70 years). Among MPAL cases median hemoglobin (Hb) level was 7g/dl (range: 5.2-10.8 g/dl), median platelet count was 42×109/L (range: 4 – 292 ×10^9^/L) and median white blood cell (WBC) count was 14×10^3^ (range: 05-215 ×10^3^). Median neutrophil count was 1100 cells/µL (range: 1-1170 cells/µL) and median blast cells was 48%. Lactate dehydrogenase (LDH) level was higher (median 265 U/L, range: 105-1298 U/L) (Supplementary Table- 1).

### 3.3 Morphological and immunophenotypic profile of 22 patients with mixed-phenotype acute leukemia (MPAL)

The baseline morphological and immunophenotypic characteristics of the 22 patients included in the study are summarized (Table-2 and Supplementary Table- 2). Of the total cohort, 15(68.2%) patients were morphologically diagnosed with acute myeloid leukemia (AML) and 7 (31.8%) with acute lymphoblastic leukemia (ALL). Peripheral blood blast percentages showed wide variability across the study population, with values ranging from 15% to 85%. Overall, the mean blast percentage was 45.4 ± 22.6%.

**Table 2:** Morphological and immunophenotypic profile of patients with mixed-phenotype acute leukemia (*n* = 22).

Variable	MPAL (*n* = 22)	AML (*n* = 15)	ALL (*n* = 7)	*p*-value
**Blast in peripheral blood (%)**				
Mean ± SD	45.4 ± 22.6	51.7 ± 22.5	33.4 ± 21.3	**0.048**†
Median (Range)	48 (15–85)	53 (15–85)	25 (15–57)	
**Blast morphology**				
Homogenous	10 (45.5%)	4 (26.7%)	6 (85.7%)	**0.03‡**
Heterogenous	12 (54.5%)	11 (73.3%)	1 (14.3%)	
**Immunophenotype**				
B/Myeloid	15 (68.2%)	12 (80%)	3 (42.8%)	—
T/Myeloid	5 (22.7%)	3 (20%)	2 (28.6%)	—
B/T	2 (9.1%)	0	2 (28.6%)	—

† Mann–Whitney U test.

‡ Fisher’s exact test.

— Not tested due to small subgroup sizes.

Regarding blast morphology, heterogenous blast populations were more frequently observed (54.5%) than homogenous populations (45.5%) in the overall cohort. AML cases showed higher blast percentages compared to ALL cases (*p* = 0.048) (Fig. 1).

**Fig. 1:**
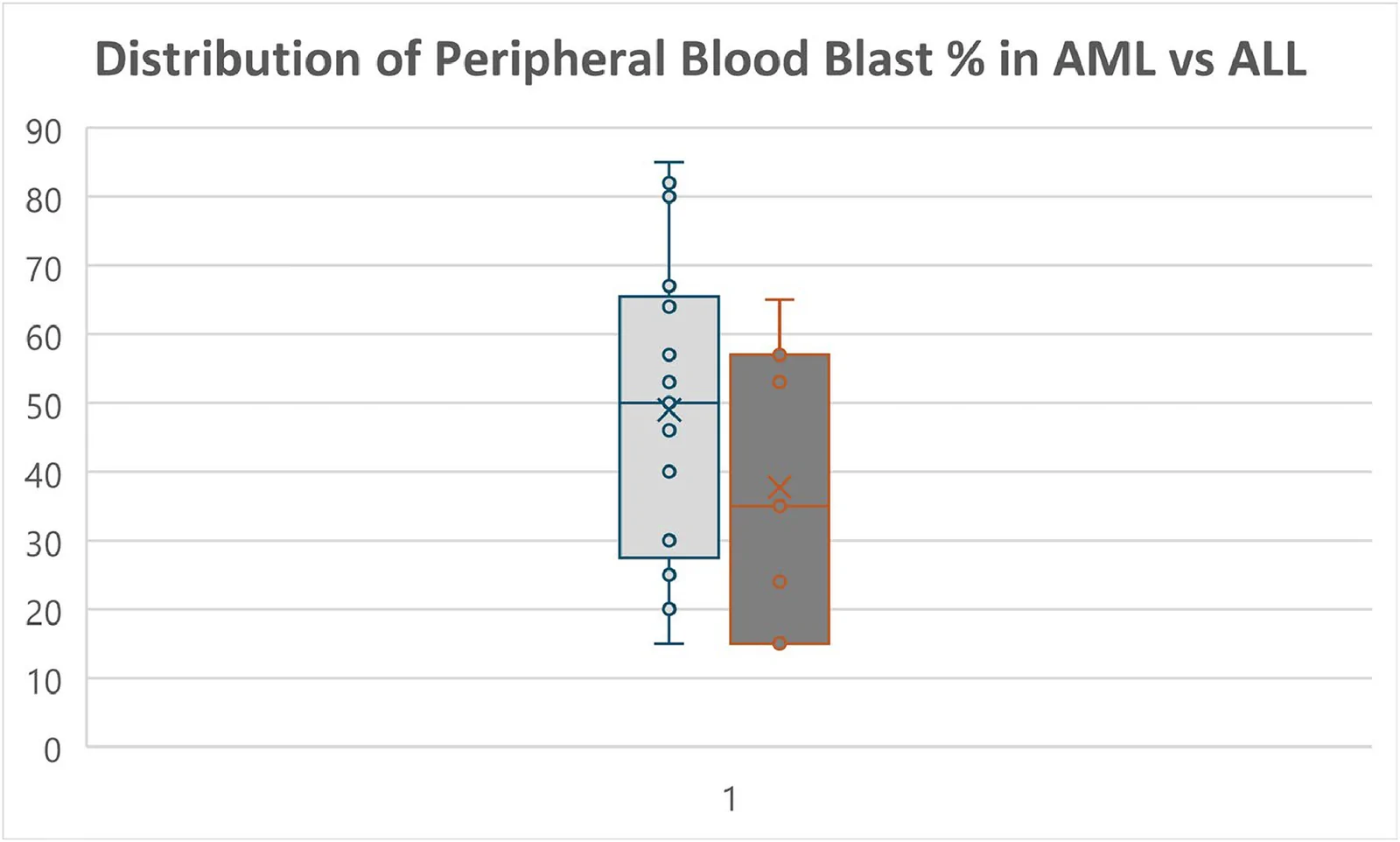
Distribution of peripheral blood blast percentage in AML and ALL. Box-and-whisker plots demonstrate the median, interquartile range, and range of peripheral blood blast percentages among AML and ALL patients. AML cases showed higher blast percentages compared to ALL cases (Mann–Whitney U test, *p* = 0.048).

Among AML patients, heterogenous morphology predominated, whereas ALL cases showed a higher proportion of homogenous blast populations. Although heterogenous blasts were more frequent in AML, the difference was statistically significant (*p* = 0.03) (Fig. 2).

**Fig. 2:**
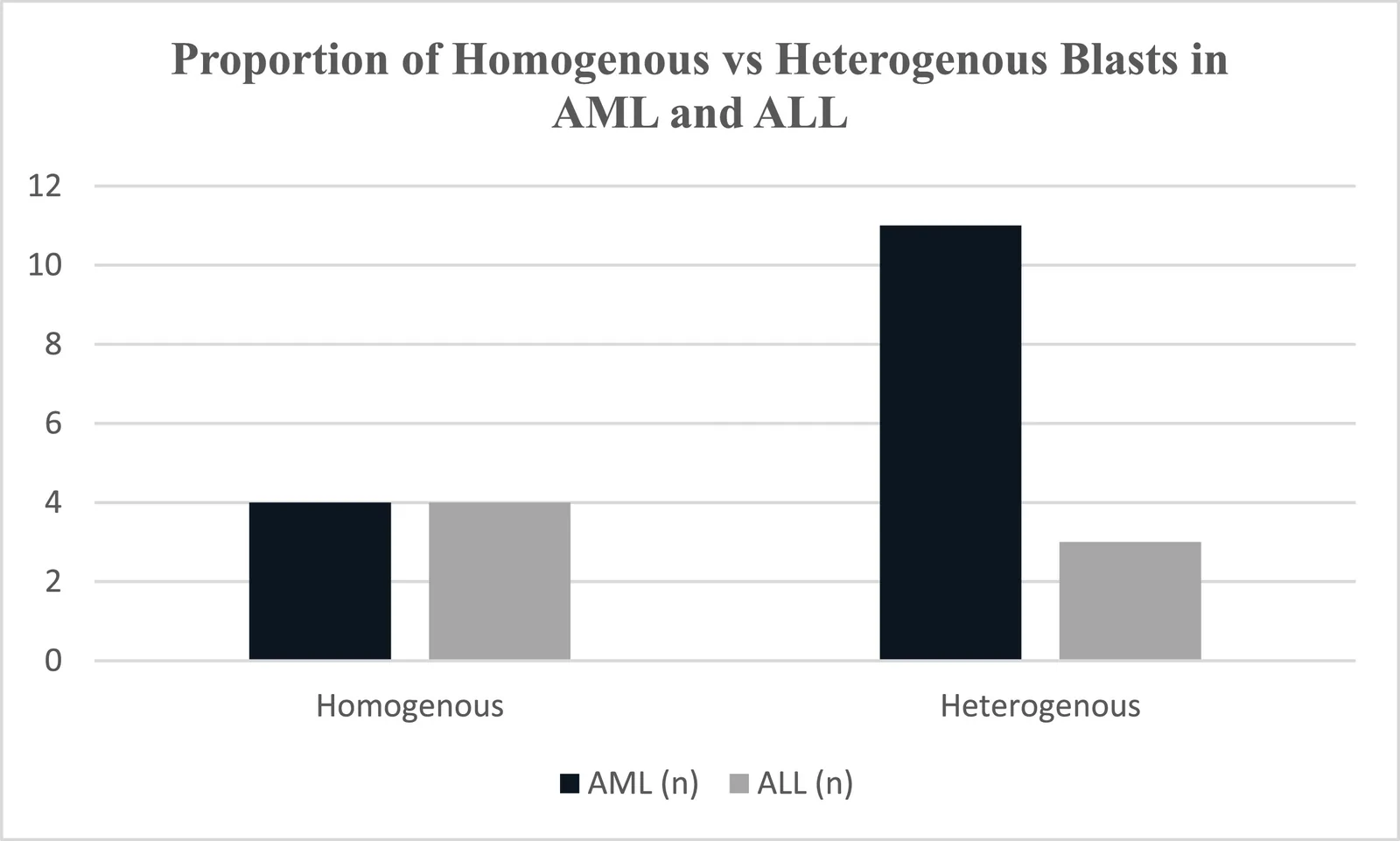
Distribution of blast morphology in AML and ALL. The bar chart demonstrates the proportion of homogenous and heterogenous blast populations among AML and ALL patients. Although heterogenous blasts were more frequent in AML, the difference was statistically significant (Fisher’s exact test, *p* = 0.03).

Immunophenotypic analysis revealed that mixed phenotype expression was common. The B/Myeloid immunophenotype represented the largest subgroup, accounting for 68.2% of all cases, followed by T/Myeloid (22.7%) and B/T (9.1%). The predominance of mixed lineage immunophenotypes was more evident among AML cases, while B/T immunophenotype was observed exclusively in ALL patients (Table 2).

All 22 patients with MPAL were morphologically analyzed according to the French- American- British (FAB) criteria [6]. Morphological characteristics of the blasts are summarized in Supplementary Table -2 and representative microscopic features of three cases of MPAL has been shown in Supplementary Figs. 2(A), (B) and (C). Of 22 MPAL cases, 15 (68.2%) were morphologically diagnosed as acute myeloid leukemia (AML) namely M1, M2 and M5 according to FAB; no case was M3, M4, M6 or M7. Immunophenotypically 12/15(80%) cases were B/Myeloid MPAL and 3/15(20%) cases were T/Myeloid MPAL (Table 2).

Seven (31.8%) were classified morphologically as acute lymphoid leukemia (ALL) cases namely ALL-1 according to FAB; no case was L-2 and L-3. Immunophenotypically 3/7(42.8%) cases were B/Myeloid MPAL and 2/7(28.6%) cases were T/Myeloid MPAL and 2/7(28.6%) were B/T MPAL. Twelve of 22 cases showed heterogenous blast populations with small and large blasts whereas 10 cases showed homogenous blasts. Blasts in the periphery varies from 15- 85% (Table-2).

Supplementary Table- 2 presents the distribution of French–American–British (FAB) subtypes among the acute leukemia cases included in the study. Among patients diagnosed with acute myeloid leukemia (AML), the most frequently observed FAB subtype was AML-M2, followed by AML-M5 and AML-M1. These subtypes together accounted for all AML cases in the cohort.

All patients with acute lymphoblastic leukemia (ALL) demonstrated L1 morphology according to the FAB classification. Due to the limited number of cases within each FAB subtype, the analysis of FAB classification was descriptive in nature, and no inferential statistical comparisons were performed.

### 3.4 Immunophenotypic expression profile of B/myeloid, T/myeloid and B/T cases

The cases were diagnosed as Mixed-phenotype acute leukemia (MPAL) as per the World Health Organization (WHO) 2008 criteria [3].

Immunophenotyping showed that out of 22 MPAL cases 15 (68.2%; 95% CI: 45.1–86.1%) cases were B-lymphoid + myeloid immunophenotype (B/Myeloid), 5 (22.7%; 95% CI: 7.8–45.4%) cases were T-lymphoid+myeloid immunophenotype (T/Myeloid) and 2 (9.1%; 95% CI: 1.1–29.2%) cases were B-lymphoid+T-lymphoid immunophenotype (B/T) MPAL (Table 1; Supplementary Fig. 1).

#### 3.4.1 Expression of myeloid lineage markers in B/Myeloid and T/Myeloid immunophenotype

The detection of cytoplasmic myeloperoxidase (cyMPO) by flowcytometry with an anti-MPO antibody is considered the most significant marker. The anti-MPO was expressed in all B/Myeloid and T/Myeloid MPAL cases. Other myeloid markers were CD13, CD33, CD117 also expressed (Supplementary Table-4; Fig. 3, Fig. 4).

**Fig. 3:**
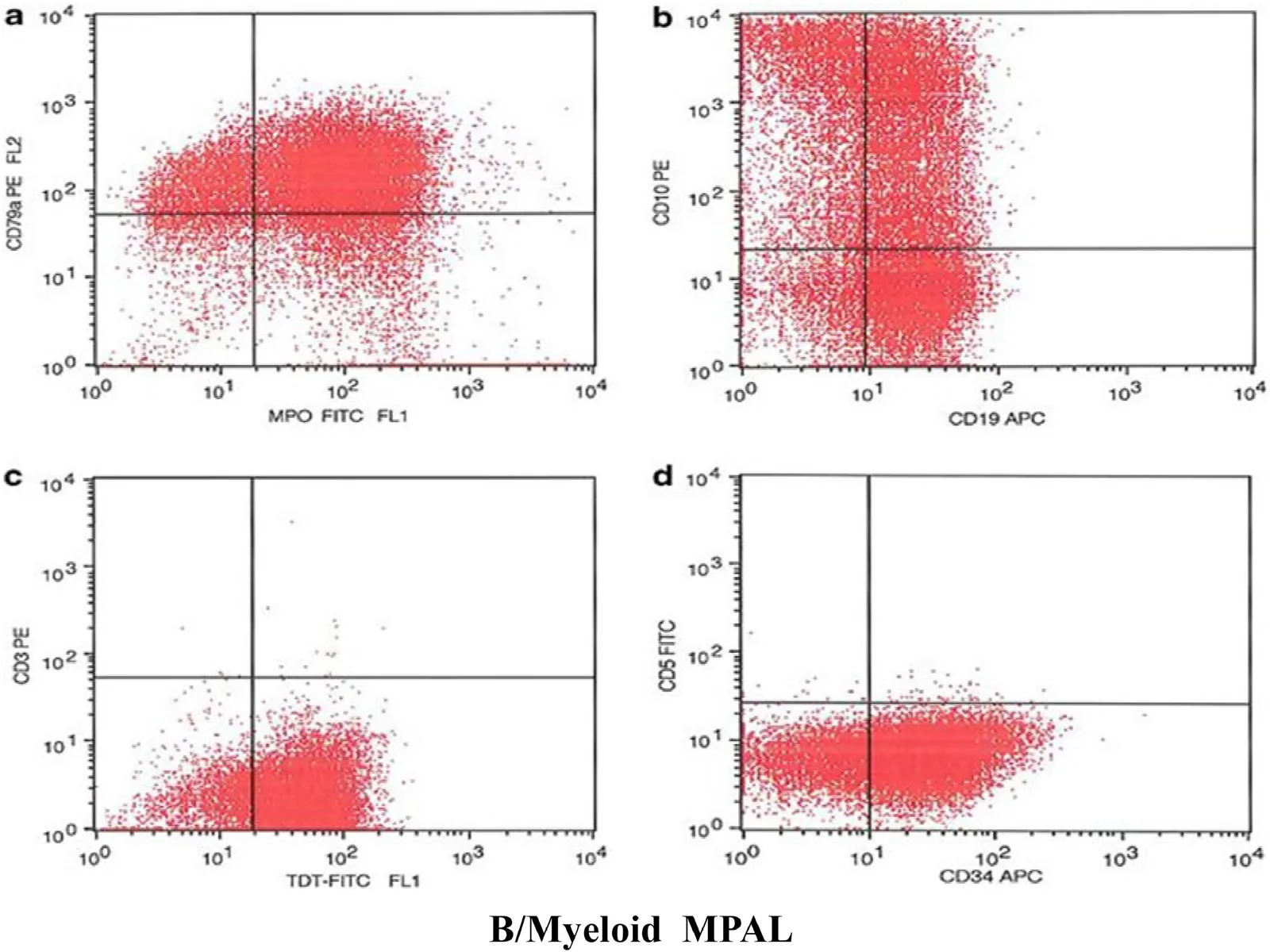
Dot Plot of a case of B/Myeloid mixed phenotype acute leukemia. The (a) scattergram showing population of cells coexpressing both CD79a (B cells) and MPO (myeloid cells) indicating B/Myeloid MPAL. These cells are also expressing CD19 and CD10, TdT and CD 34 in **(b), (c) and (d)** scattergram respectively.

**Fig. 4:**
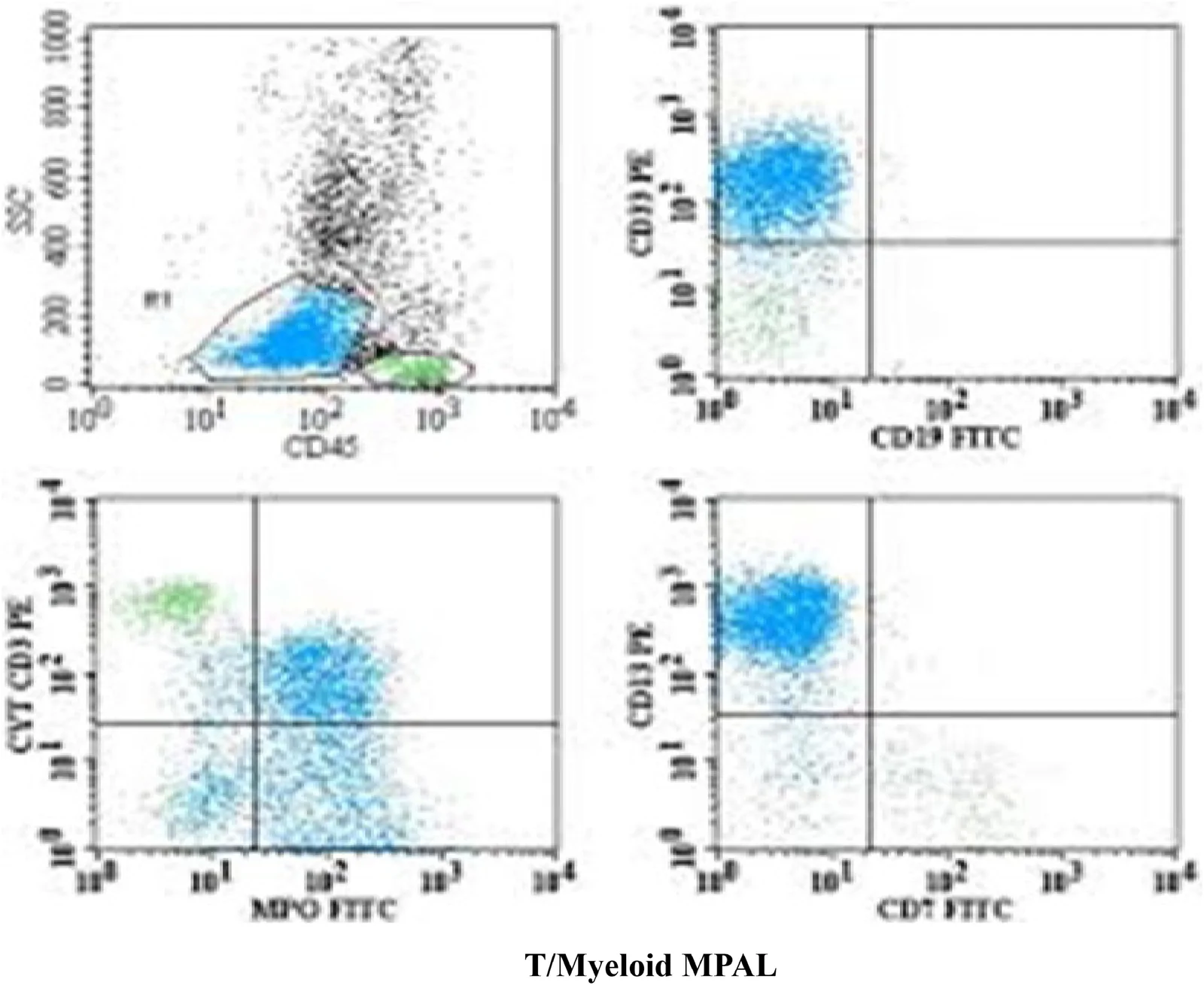
Dot Plot of a case of T/Myeloid Mixed phenotype acute leukemia. The 3rd scattergram showing population of cells coexpressing both CD3 (T cells) and MPO (myeloid cells) indicating T/Myeloid MPAL. These cells are also expressing CD33 and CD13 in 2nd and 4th scattergram respectively.

#### 3.4.2 Expression of T-lymphoid lineage markers in T/Myeloid immunophenotype

All T/Myeloid cases expressed T-lymphoid lineage marker cytoplasmic CD3 (cyt CD3), had more than 20% cytCD3+ blasts and coexpressed with anti-MPO. Associated T- lymphoid lineage markers CD5, CD7 were also expressed. There were expression of immaturity markers CD34, TdT and HLA-DR (Supplementary Table-4; Fig. 4).

#### 3.4.3 Expression of B-lymphoid lineage markers in B/Myeloid immunophenotype

There were strong expression of B-lymphoid lineage markers CD19 and cytCD79a in all B/myeloid MPAL. Dim to moderate expression of CD10 and CD22 were observed. More than 20% cytCD79a+ and CD19+ blasts were coexpressed with anti-MPO. Expression of immaturity markers CD34, TdT and HLA-DR were observed (Supplementary Table-4; Fig. 3).

#### 3.4.4 Expression of both B-lymphoid and T-lymphoid lineage markers in B/T MPAL immunophenotype

More than 20% blasts showed strong expression of B-lymphoid lineage markers CD19 and cytCD79a with coexpression of T-lymphoid lineage marker cyt CD3. Other B-lymphoid markers CD10, CD22 and T-lymphoid markers CD5 and CD7 were expressed. Markers of immaturity CD34, TdT and HLA-DR were observed (Supplementary Table-4; Fig. 5).

**Fig. 5:**
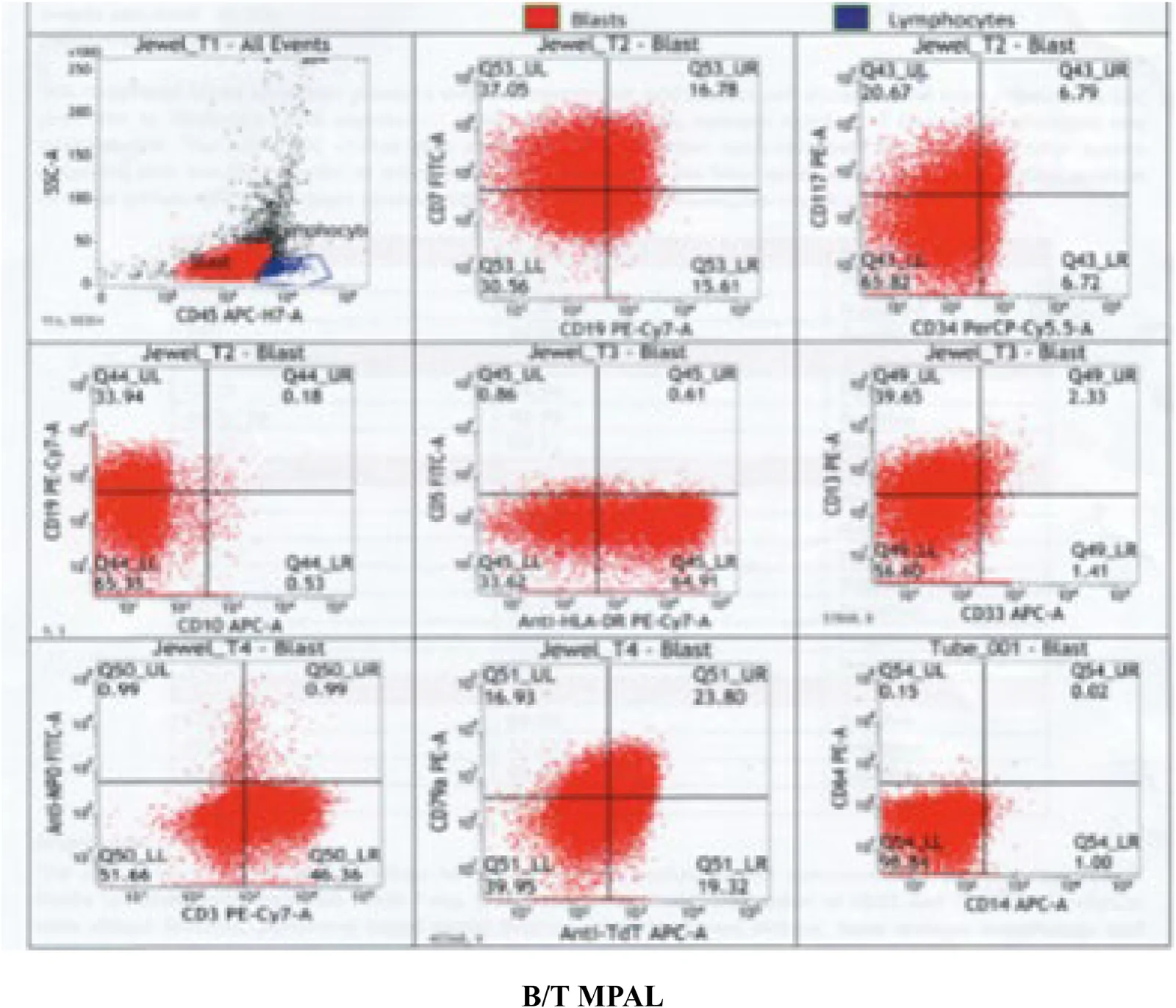
Dot Plot of a case of B/T Mixed phenotype acute leukemia. The lower 7th and 8th scattergram coexpressing both CD3 (T cell) and CD79a (B cell) indicating B/T MPAL. Other scattergrams also showing expression of CD19, CD7, HLA-DR, TdT.

## 4 Discussion

Before WHO 2008 classification system, the diagnosis of MPAL was mostly based on scoring systems which was described by Catovsky et al. [7] in 1991 and was followed by European Group for the Immunological Classification of Leukemias (EGIL) criteria in 1995 [8]. St. Jude's criteria for the diagnosis of MPALs for the first time utilized a combination of lineage-specific markers without scores to diagnose MPALs [9]. The frequency of MPAL was 3.57% in this study and was similar (2.4%) to that seen by several studies [[10], [11], [12]]. The median age of MPAL patients in this study was 23.5 years (range, 1-70 years), indicating that MPAL occurred both in children and adults. There was an overall male predominance, with 16 males and 6 females (male-to-female ratio approximately 2.7:1). The male-to-female ratio was 5:2 among adults and 3:1 among children. Although male predominance has also been reported in other studies, this observation should be interpreted cautiously because of the small number of cases in the present cohort [4,11]. Male predominance may be due to social factors leading to more attendance of male patients in the hospital. Immunophenotypic subtypes did not show any correlation with age or sex. The analysis of immunophenotypic profile of the MPAL cases in this study revealed 15 (68.18%) B/myeloid, 5(22.72%) T/myeloid and 2 (9.1%) as B/T MPAL cases. A study showed similar findings where out of 117 MPAL cases, 64 (55%) were B/myeloid, 38 (33%) were T/myeloid, 14 (12%) were B/T lymphoid and one (0.9%) case was trilineage MPAL [12].Another study observed similar findings [11]. Overall, B/myeloid MPAL appears to be the more common subtype; however, analysis of a greater number of patients would be required to confirm it. MPAL cases were diagnosed morphologically as 15 acute myeloid leukemia (AML) and 7 acute lymphoid leukemia (ALL) in this study whereas blast morphology was not predictive of a MPAL. MPAL is morphologically heterogenous, consisting of small and large blast population which would predict the possibility of MPAL. The subgroup AML (*n* = 15) and ALL (*n* = 7) are relatively small and unequal subgroup sizes, analyses of morphological comparison of these subgroups should be considered exploratory. So, differences should not be considered definitive but should be interpreted cautiously . These findings are hypothesis-generating and require larger, multicenter studies for confirmation.

Most of the 12 B/Myeloid MPAL cases were morphologically AML-M1 or M2 or M5 and three cases were ALL-L1 whereas 2 T/Myeloid cases were ALL-L1 and 3 were AML-M1, M2 and M5. Two B/T MPAL case were ALL-L1. Almost similar findings reported by Matutes et al. [4].

In leukemias aberrant expression of antigens of one or more lineage markers is common [3]. So, acute leukemias with aberrant antigen expression should be differentiated from MPAL for a definitive diagnosis. According to 2008 WHO classification, expression of myeloperoxidase (MPO) or expression of at least two monocytic markers define the myeloid lineage assignment; expression of cytoplasmic or surface CD3 define T-cell lineage commitment; B-lineage commitment is defined by expression of either strong CD19 and strong expression of at least one of the following: CD79a, cytoplasmic CD22, CD10 or weak CD19 and strong expression of at least two of the following: CD79a, cytoplasmic CD22, CD10 [3].

In this study, MPO positivity for myeloid lineage identification, for B-lymphoid lineage CD19, CD79a, CD22 and cyCD3 or surface CD3 for T-lymphoid lineage on flow cytometry were used in categorizing a case as MPAL. It is necessary to standardize sample processing carefully as MPO positivity is critical for identification of MPAL.

Cell of origin of MPAL is still not known but it is thought that this leukemia arises from a multipotential hemopoietic stem cell with potential to differentiate into either myeloid or lymphoid lineage or rarely B-cell and T-cell lineage. Most of the reported cases of MPAL express early hematopoietic markers-CD34, HLA-DR and TdT suggesting an early precursor stem cell origin [13]. Expression of immaturity markers CD34, TdT and HLA-DR were observed in this study which reinforces the early stage of differentiation of blasts.

The primary objective of this retrospective study was to characterize morphologically diagnosed acute leukemia cases by multiparametric flow cytometry and diagnose MPAL following the WHO 2008 criteria. This study did not assess overall survival, treatment response, relapse, measurable residual disease (MRD). The present dataset would not be able to determine therapeutic and prognostic implications of MPAL. Outcomes demonstrated by the present investigation did not imply poor outcome and potential treatment of MPAL and that should be regarded as background information derived from previous studies.

Mixed phenotype acute leukemia (MPAL) are rare leukemias with a poor clinical outcome. It should be differentiated from acute leukemias with aberrant antigen expression. Accurate diagnostic classification of MPAL is important because morphology alone may not reliably identify cases with mixed lineage characteristics. Therefore, contribution of this study is the identification and immunophenotypic characterization of MPAL among morphologically diagnosed acute leukemia cases using flow cytometry.

## 5 Limitations

This study has several limitations. First, The morphological diagnosis of acute leukemia in the present study was done according to the French-American-British (FAB) classification during the original clinical evaluation. The present study could not apply contemporary molecularly integrated classification as this study was conducted retrospectively using cases between 2015 and 2019, where comprehensive molecular and cytogenetic data were not available. Therefore, retrospective reclassification was not feasible.

Second, MPAL cases were classified according to the World Health Organization (WHO) 2008 classification criteria, which were applicable during the study period (November 2015 to December 2019). For all patients molecular and cytogenetic investigations were not available. Therefore, specific genetic abnormalities such as BCR::ABL1 or KMT2A rearrangements, could not be retrospectively assigned in this study.

Third, the study was performed during 2015-2019 using a four-color platform, the methodology could not incorporate contemporary 8-10 color or higher-color flow cytometry because of its unavailability, which may limit the ability to distinguish true multilineage differentiation from aberrant antigen expression.

Fourth, this study did not assess overall survival, treatment response, relapse, measurable residual disease (MRD). The present dataset would not be able to determine therapeutic and prognostic implications of MPAL.

Fifth, the subgroup AML (*n* = 15) and ALL (*n* = 7) are relatively small and unequal subgroup sizes, analyses of morphological comparison of these subgroups should be considered exploratory. So, differences should not be considered definitive but should be interpreted cautiously . The number of confirmed MPAL cases was relatively small (*n* = 22), resulting in limited statistical power and wide confidence intervals around several subtype estimates.

Finally, this was a single-center study conducted in a tertiary referral setting, further larger prospective multicenter studies incorporating cytogenetic and molecular investigations together with contemporary multiparametric flow cytometry and longitudinal clinical follow-up are warranted.

## 6 Conclusion

Morphologically diagnosed Acute leukemia cases can be accurately diagnosed as MPAL by flowcytometric immunophenotyping. Multiparametric flow cytometry using comprehensive panel of monoclonal antibodies is a valuable diagnostic tool for identifying MPAL, as it is a rare immunophenotypically heterogeneous subset of acute leukemia. As far as we know, this is the first report of flow cytometric immunophenotypically diagnosed MPAL in Bangladesh. The findings of this study highlight the value of multiparametric flow cytometry to diagnose MPAL in peripheral blood or bone marrow specimens that can be missed out by morphology alone. To determine the prognostic and therapeutic implications of MPAL in the Bangladeshi population further prospective studies incorporating cytogenetic and molecular findings, treatment response, survival, relapse and MRD assessment are needed.

## Submission declaration and verification

This manuscript has not been published and is not under consideration for publication elsewhere.

## Previous presentations

6th European Congress of Immunology (ECI) 2021:

Tarafder S, “Mixed-phenotype acute leukemia: Flow cytometric analysis in a tertiary care center of Bangladesh” Poster presentation No. 0090, September 1-4, 2021.

BSBMB-SABC-OMC International Conference 2023:

Tarafder S, “Mixed-phenotype acute leukemia: Flow cytometric analysis in a tertiary care center of Bangladesh” Oral presentation, February 3-5, 2023.

## Ethics declaration

Written informed consent to take part in the study and to publish the article has been obtained from all participants or their legal representatives. The privacy rights of participants have been observed.

This study was performed in compliance with relevant laws, regulatory frameworks and guidelines where the research took place. This study was approved by the Bangladesh Bioethics Society. (Approval No. Ref: ERC/BBS/27/2022 Date: 10. 04. 2022).

## Funding

This research did not receive any specific grant from funding agencies in the public, commercial, or not-for-profit sectors.

## CRediT authorship contribution statement

**Shirin Tarafder:** Writing – review & editing, Validation, Supervision, Methodology, Investigation, Conceptualization. **Sheikh Anisul Haque:** Writing – original draft, Validation, Software, Methodology, Investigation, Data curation.

## Declaration of competing interest

The authors declare that they have no competing financial interests or personal relationships that could have appeared to influence the work reported in this paper.
