# Supplementary material for: Mixed-phenotype acute leukemia: Flow cytometric analysis in a tertiary care center of Bangladesh
Source: Leuk Res Rep. 2026 Sep 23;26:100612. doi: 10.1016/j.lrr.2026.100612 (PMC13639598; doi:10.1016/j.lrr.2026.100612)
Supplement: Supplementary file 1 [file mmc1.docx]

**Appendices (Supplementary Material):**

**The following are the supplementary data related to this article:**

**APPENDIX- I**

**Distribution of Mixed-Phenotype acute leukemia (MPAL) cases (n=22)**

**Immunophenotypic diagnosis of 616 cases**

|  |  |
| --- | --- |
| Acute Leukemia | 96.43 |
| MPAL | 3.57 |

|  |  |
| --- | --- |
| B/Myeloid | 68.18 |
| T/Myeloid | 22.72 |
| B/T | 9.1 |

**Supplementary Figure 1: Immunophenotypic diagnosis and distribution of Acute leukemia and Mixed phenotype acute leukemia cases**

**Supplementary Figure -2.(A)** A case of B/Myeloid MPAL showing a heterogenous population of small and large blasts. **(B)** A case of T/Myeloid MPAL showing small to intermediate blasts. **(C)** A case of B/T MPAL showing homogenous blast population.

**Supplementary Table 1: Patient characteristics**

|  | Number (%) |
| --- | --- |
| Gender  Male  Female | 16/22 (72.73)  6/22 (27.27) |
| Immunophenotype  B/Myeloid  T/Myeloid  B/T | 15/22 (68.18)  5/22 (22.72)  2/22 (9.1) |
|  | Median(range) |
| Age (years) | 23.5 years ( 1-70 years) |
| WBC (×10^3^). | 14 ( 05- 215) |
| Platelets (ˣ10 ^9^/L) | 42 ( 4- 292) |
| Hemoglobin (g/dl) | 7 ( 5.2- 10.8) |
| Total neutrophil count (cell/µL) | 1100 ( 1- 1170) |
| Blast cells (%) | 48% ( 15-85) |
| LDH (U/L) | 265 (105- 1298) |

**Supplementary Table 2: Morphological and immunophenotypic profile of 22 patients with mixed-phenotype acute leukemia**

| Patient no. | Blast in periphery (%) | Type of blast populations  (morphology) | FAB subtype  AML(n=15)  ALL(n=7) | Immunophenotype |
| --- | --- | --- | --- | --- |
| 1 | 40 | Heterogenous | AML-M5 | B/Myeloid |
| 2 | 65 | Homogenous | ALL-L1 | T/Myeloid |
| 3 | 80 | Heterogenous | AML-M2 | B/Myeloid |
| 4 | 25 | Homogenous | AML-M5 | B/Myeloid |
| 5 | 57 | Homogenous | AML-M1 | T/Myeloid |
| 6 | 21 | Heterogenous | AML-M1 | B/Myeloid |
| 7 | 50 | Heterogenous | AML-M2 | T/Myeloid |
| 8 | 82 | Heterogenous | AML-M2 | B/Myeloid |
| 9 | 30 | Homogenous | AML-M5 | T/Myeloid |
| 10 | 15 | Homogenous | ALL-L1 | B/Myeloid |
| 11 | 53 | Heterogenous | AML-M1 | B/Myeloid |
| 12 | 35 | Homogenous | ALL-L1 | B/T |

| Patient no. | Blast in Periphery (%) | Type of blast populations(morphology) | FAB subtype  AML(n=15)  ALL(n=7) | Immunophenotype |
| --- | --- | --- | --- | --- |
| 13 | 67 | Heterogenous | AML-M2 | B/myeloid |
| 14 | 46 | Heterogenous | AML-M1 | B/myeloid |
| 15 | 85 | Heterogenous | AML-M5 | B/myeloid |
| 16 | 57 | Homogenous | ALL-L1 | B/Myeloid |
| 17 | 53 | Homogenous | ALL-L1 | T/Myeloid |
| 18 | 15 | Heterogenous | AML-M2 | B/myeloid |
| 19 | 20 | Heterogenous | AML-M2 | B/myeloid |
| 20 | 24 | Homogenous | ALL-L1 | B/T |
| 21 | 15 | Heterogenous | ALL-L1 | B/Myeloid |
| 22 | 64 | Homogenous | AML-M5 | B/Myeloid |

**Supplementary Table 3. FAB Subtype Distribution**

| **FAB Subtype** | **Number (%)** |
| --- | --- |
| AML-M2 | 6 (40.0%) |
| AML-M5 | 5 (33.3%) |
| AML-M1 | 4 (26.7%) |
| ALL-L1 | 7 (100% of ALL) |

**Supplementary Table 4: Immunophenotypic expression profile of B/myeloid, T/myeloid and B/T cases**

| **Types of MPAL** | **Markers** |
| --- | --- |
| B/Myeloid (n=15) | Cy MPO, CD13, CD33, CD117  CD19 ^strong^, cyCD79a ^strong^, CD22, CD10 ^dim/moderate^  CD34, HLA-DR, TdT |
| T/Myeloid (n=5) | Cy MPO, CD13, CD33, CD117  cyCD3, CD5, CD7  CD34, HLA-DR, TdT |
| B/T (n=2) | CD19 ^strong^, cyCD79a ^strong^, CD22, CD10 ^dim/moderate^  cyCD3, CD5, CD7  CD34, HLA-DR, TdT |

**Supplementary Table-5. 2008 WHO classification of acute leukemias of ambiguous lineage ^14^**

**Supplementary Table-6.** **The European Group for the Immunological Classification of Leukemias (EGIL) scoring system ^8^**

Abbreviations: MPO, myeloperoxidase; TdT, terminal deoxynucleotidyl transferase; cyt, cytoplasmic; TCR, T-cell receptor. ≥ 2 points are required to assign a lineage.

**APPENDIX- II**

**WORKFLOW CHART**

Study population (616 patients)

Data were extracted from the database and anonymized, whose peripheral blood or bone marrow specimens were submitted for MFC immunophenotyping and cell morphology using Giemsa stain

The morphological diagnosis were incorporated with MFC analysis, reviewed and categorized them into Mixed-phenotype acute leukemia (MPAL) or acute leukemia categorized by WHO 2008 criteria

.

Data were verified and crosschecked, Report writing

Study outcome:

- Evaluated the analysis outcome
- Suggesting helpful assessment techniques for future patients.
